# Nature in the Heart and Mind of the Beholder: Psycho-Emotional and EEG Differences in Perception of Virtual Nature Due to Gender

**DOI:** 10.3390/vision7020030

**Published:** 2023-04-03

**Authors:** Artem Davidov, Olga Razumnikova, Maxim Bakaev

**Affiliations:** Department of Data Collection and Processing Systems, Novosibirsk State Technical University, 630073 Novosibirsk, Russiarazoum@mail.ru (O.R.)

**Keywords:** virtual reality, user experience, electroencephalography, psychometric data, emotional state, environmental identity

## Abstract

Natural environment experiences in virtual reality (VR) can be a feasible option for people unable to connect with real nature. Existing research mostly focuses on health and emotional advantages of the “virtual nature” therapy, but studies of its neuropsychological effects related to visual perception are rare. In our experiment, 20 subjects watched nature-related video content in VR headsets (3D condition) and on a computer screen (2D condition). In addition to the gender factor, we considered the individual Environmental Identity Index (EID) and collected the self-assessment of the emotional state per the components of Valence, Arousal, and Dominance in each experimental condition. Besides the psychometric data, we also registered brainwave activity (EEG) and analyzed it with the 7 frequency bands. For EID, which was considerably higher in women, we found significant positive correlation with Valence (i.e., beneficial effect of the natural stimuli on the psycho-emotional status). At the same time, the analysis of the EEG data suggests a considerable impact of the VR immersion itself, with higher relaxation alpha effect in 3D vs. 2D condition in men. The novel and most pronounced effect of the gender factor was found in the relation between the EID and the EEG powers in the high-frequency bands—that is, positive correlation of these variables in women (0.64 < Rs < 0.74) but negative correlation in men (−0.66 < Rs < −0.72). Our results imply individually different and gender-dependent effects of the natural stimulus in VR. Correspondingly, the video and VR content development should consider this and aim to provide a user characteristics-tailored experience.

## 1. Introduction

The development of Virtual Reality (VR) technologies has led to their intense application in various fields of psychotherapy, including stress-related problems (e.g., see [1,2]). The purposeful design of the virtual environment allows for the creation of relevant conditions for studying individual emotional reactions to the particular sensory stimuli, foremost visual ones. Particularly, increased urbanization and an accelerating pace of life make contacts of city dwellers with nature more problematic, which provided the boost for the “digital eco-therapy”—the dedicated interaction with virtual nature. This trend is also consistent with the current sustainable development goals and the emphasis made on health care and well-being support. However, due to the general enthusiasm in promoting VR/AR, its actual impact, particularly on inexperienced user groups, is not explored as much [3]. Existing research in virtual eco-therapy mostly relies on surveys and interviews, which are inherently subjective and do not provide deep insights into visual perception.

Meanwhile, the diversity of individual responses to VR immersion is high, and there is a possibility of negative effects, such as dizziness, headaches, perceptual-motor disturbances, or emotional tension [4,5,6,7,8]. The combination of psychometric measures and EEG analysis has revealed the lack of concordance in them with respect to experience from experimental videos. Existing research notes restoration of hedonic tone and stress reduction as a result of viewing historical elements in the architecture of the city without a significant effect on self-reported mental state of the presence of greenery, which, however, was accompanied by lower levels of alpha activity in the occipital regions of the brain as a reflection of visual attention [9]. Correspondingly, although VR technologies have been highly praised, their final effectiveness for cognitive training or emotional healing is not guaranteed and deserves fair-minded studies.

Therefore, the goal of our research work was to investigate the changes in emotional self-assessment and analyze the activity of the cerebral cortex in terms of the power of low-frequency and high-frequency EEG rhythms upon presentation of images of the natural environment in 2D and 3D format. In this, we explore the effect of the subjects’ gender on their emotional and spatial perception of the natural landscape. In the sample of 20 subjects of different genders, we considered the individual environmental identity, measured the emotional status using self-assessment, and registered EEG signals in the 7 frequency bands. We believe that the main contribution of our study is the discovered individual differences in the self-assessment of emotional reactions to biophilic stimuli and the reflection of their perception in the brain activity. These might be applicable in the development of eco-therapy programs for improving emotional conditions and alleviating cognitive deficits. The preliminary results of our project were presented in the Problems of Informatics, Electronics, and Radio Engineering (PIERE 2022) international conference in Nov 2022.

The remainder of our work is structured as follows. In Section 2, we briefly outline related work in the field. In Section 3, we describe the experiment and specify the hypotheses. In Section 4, we analyze the psychometric and the electroencephalographic data for the different experimental conditions and the user groups. In Section 5, we summarize our conclusions and outline directions for further research.

## 2. Methods and Related Work

Existing studies suggest that city dwellers regularly experience stress since their life is especially prone to threats to both physical and mental well-being. Quality of habitat, neighbors, various noises, polluted or stale air, unnatural lighting—all of these affect psychological health [10]. Adaptation mechanisms become exhausted, and stress-related responses in their body and neural connections in their brain become a routine. Whereas natural surroundings, such as a riverside, cause positive emotional reactions due to our evolutionary history since they enhance a human’s survival capabilities. Indeed, it has been demonstrated that watching landscapes and being in contact with nature can decrease stress due to unconditioned physiological and psychological reactions [11].

VR/AR technologies are capable of providing indirect engagement with nature. They can immerse a human into visual space with pseudo-ability to move, but they do not currently provide smells, tactile impressions, etc. Still, it has been shown that VR/AR users can experience a physical presence in a location due to engagement of several feelings, including vision, hearing, and vestibular apparatus [12].

One of the popular applications of the “virtual nature” therapy is for curing loneliness and age-related dementia. Despite existing limitations, VR/AR can provide new emotions, which generally improve physical and mental state of the people. In a recent experiment in the field, the subjects were either “taken” on a virtual tour in Banf natural park or in Barcelona city. The subsequent survey demonstrated that even such short-term “contact” with nature had directly improved the psychological state, which can be well explained (e.g., by the attention recovery theory) [13].

To register the emotions and physiological arousal levels caused by the VR immersion, both self-assessment and objective psychophysiological measurements, such as electroencephalography (EEG), functional Magnetic Resonance Imaging (fMRI), electro-dermal activity (EDA), and ECG (Electrocardiogram) are commonly used [6,7,14,15,16]. These might be purposefully manipulated by virtual–visual stimuli, such as natural, urban, or industrial landscape environments [17,18,19]. Indeed, such 3D scenarios are increasingly used for the analysis of functional changes in the brain structures’ activity associated with the emotional states caused in an experiment [7,14]. Location-based analysis can also yield notable results—for instance, it has been shown that urban surroundings have caused increased activity in the amygdala, which is associated with impulsivity, anxiety, and increased stress [20].

There is a belief that 3D objects gain priority compared to 2D images in detection, attracting attention and memorization, but only if the objects have a significance for the perceiver [21]. At the same time, the connection between the degree of the immersion to the virtual environment and the emotional response, particular arousal—its major component—is noted [22]. Considering at the same time that two-eye coordination in establishing the joint 3D representation is imperfect in 2–4% of the population [23], the conclusion is that individual reactions to the VR environment needs to be studied using both subjective assessments and indexes of functional activity of the brain visual system and the emotional regulation of the cognitive processes.

In this, the analysis of gender-dependent differences in perception of 2D and 3D images is of particular interest. On the one hand, obvious disparities in spatial orientation, with more diverse strategies for encoding spatial relations found in men, including Euclidian coordinates and the distance to a control point, have been discovered previously. At the same time, women rely more on identification of the navigation landmarks [24,25], with better performance in allocentric tasks rather than in egocentric ones [25,26].

On the other hand, women demonstrate higher attention towards emotions and their employment in decision-making [27,28,29], which is accompanied by greater engagement of the prefrontal and orbitofrontal areas of the cortex, amygdala, and striatum in the reassessment of stimuli and the organization of the functions of reward or punishment systems [30,31]. These differences, obtained through fMRI, are related to a wider usage of automatic regulation or cognitive control of emotions in men, but emotional regulation with engagement of positive emotions for reassessment of the negative ones with a higher involvement of the emotional structure of the brain in women.

The ability to depict an environment and orient in it is foremost supported by the functional activity of the neural networks in the hippocampus and medial prefrontal cortex [32,33], as well as wider spread neural systems that encode visual information. These systems are responsible for the ratio between depth and movement between spatial locations and for the specific configuration of elementary and Gnostic properties of the objects and the symbolic categorization of conceptual space [34,35].

It is known that a human immersion into a natural environment is accompanied by improvement of attention and mood, as well as decrease of the stress, in accordance with the Attention Restoration Theory [36] and/or Stress Reduction Theory [37]. Correspondingly, exposure to nature in VR is seen as a relaxation and psychotherapeutic rehabilitation method [38,39], although the factors behind the effectiveness of the above theories have not been finally confirmed yet [40,41]. The effect of “virtual” nature (an underwater coral reef) experienced in three different forms—2D video on a high-definition TV, 360° video VR in head-mounted display (HMD), and computer-generated VR (CG-VR) in HMD—has been analyzed in [42]. The outcome suggests the reduction in negative affect and boredom for people in health/care settings, as well as an increase in the positive affect and nature connectedness. Although the reduction in boredom has been observed in all three conditions, the effect was the most prominent between CG-VR and TV.

Each of the 7 commonly identified EEG frequency bands presumably reflects different indexes describing the emotional state. High amplitude in the Alpha band is related to lower physical arousal, whereas the decreased power in Alpha1 reflects arousal in the visual system and its sensitivity in processing of the perceived information [43,44,45], whose effectiveness is defined by the synchronization of neural networks in Alpha and Beta [44,46]. Beta rhythm is also generally related to high-level cognitive processes and attention focusing, and high stress causes increased amplitudes in this band. The low-frequency Delta and Theta bands are often seen as markers of the internal concentration of attention when processing information and of the emotional regulation when perceiving various emotion-causing stimuli [47,48]. Therefore, in our experimental study we explore and interpret the EEG signals in each band and relate them to the other variables considered in the study.

## 3. The Experiment Description

### 3.1. Material

As the base for the natural stimulus, we hand-picked a representative YouTube VR360 5K video (https://www.youtube.com/watch?v=AXJBCATPgKs (accessed on 13 March 2022)), in accordance with several criteria: no dialogues, high quality and resolution, dynamic camera, etc. The choice of the stimulus was justified by the previous findings by [49], which suggest that the most pronounced positive stress-relieving effects can be achieved if a virtual forest environment is presented in realistic graphics.

**Figure 1:**
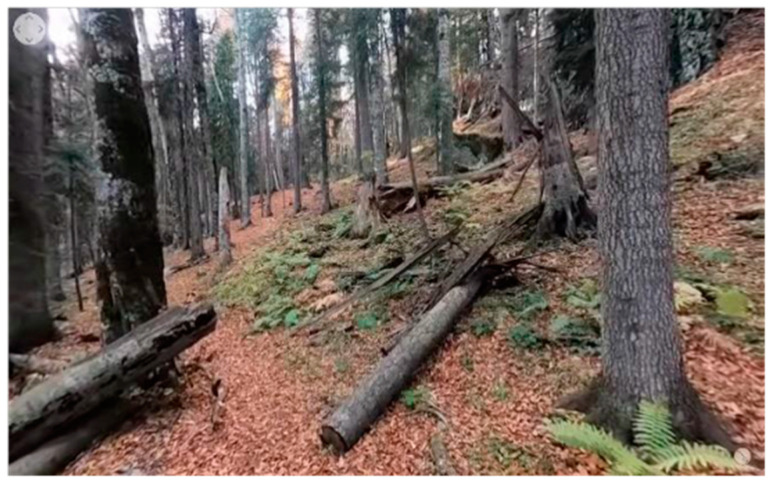
A screenshot of the “virtual forest walk” video.

The content of the video is a 2 min 3D “virtual walk” in a forest (see a representative screenshot in Figure 1). From the video, we also created a 2D version suitable for watching on a computer screen without VR equipment.

### 3.2. Subjects

**Figure 2:**
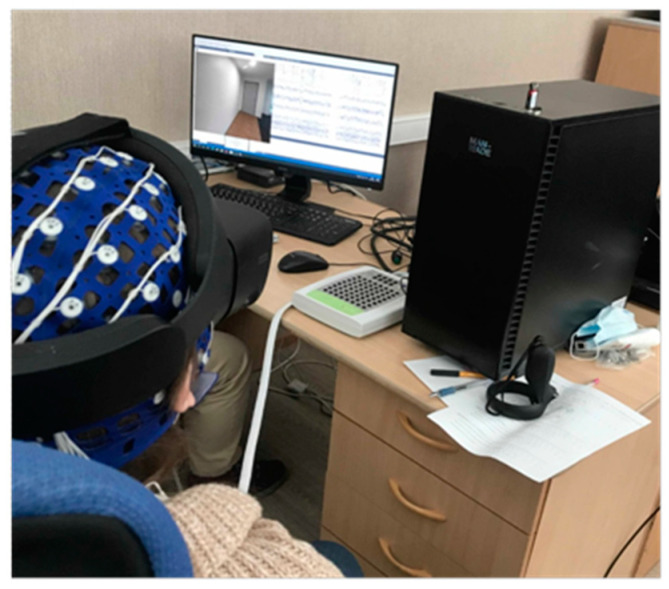
Illustration of the experimental setup (EEG plus Oculus Rift S).

The subjects were 20 students (age range 18–23, 50% men and 50% women) from the Novosibirsk State Technical University, who participated in the study voluntarily. They reported no health problems and had no prior experience of using the Oculus Rift headset. The experimental setup of the headset with the EEG cap is demonstrated in Figure 2.

All the participants were informed about the goals of the research and provided their informed consent. The research was approved by the Ethical Committee of the Faculty of Humanities of NSTU (statement No.2 of 17 February 2022).

### 3.3. Design and Procedure

Our experiment had within-subjects design, as each of the participants was subjected to the three experimental conditions, lasting 3–5 min each:

1. Control: sitting calmly with the eyes open,
2. 2D: watching the converted video on a computer screen,
3. 3D: watching the VR 360 virtual nature video (a walk in a forest).

**Figure 3:**
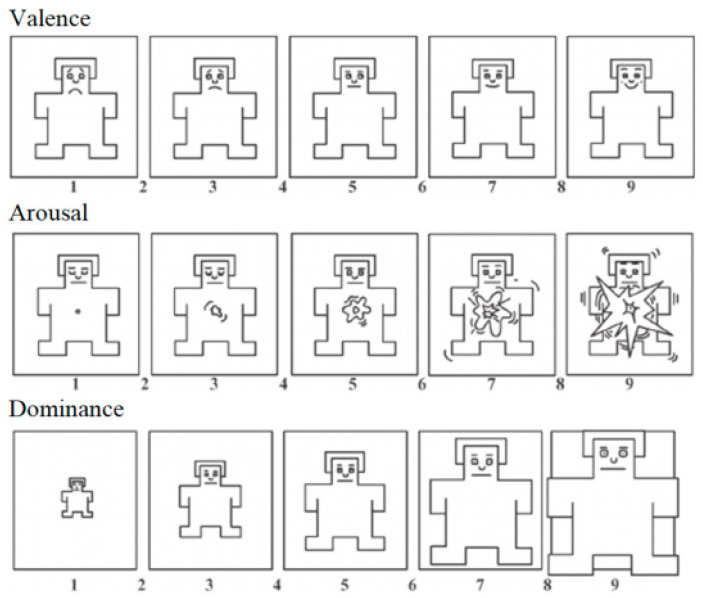
The Self-Assessment Manikin (SAM) sheet, with the scores on the three scales.

The independent variables were gender (man or woman) and the stimulus type (2D or 3D). The dependent variables were:

1. The “Environmental Identity” index (EID) [50], assessed for each subject prior to the experimental trials. We used the Russian version of the EID scale, which consists of 24 items [51]. The participants used a 5-point scale to indicate the extent to which the given statements described their general relationship with the natural environment (1 = strongly disagree, 5 = strongly agree). For example, *“I consider myself part of nature, inseparable from it”* or *“When I am sad or stressed, I helps to spend some time alone with nature”*. Correspondingly, the possible EID scores were in the range from 24 to 120, where higher values reflect the predisposition to form an emotional connection to the natural environment. The internal consistency among the items was good (Cronbach’s α = 0.88).
2. The subject’s emotional state was assessed after each of the three experimental conditions via the 3 components from the Self-Assessment Manikin (SAM) Test [52], all measured on a 9-point-likert scale (Figure 3):
  - Valence (sad–happy),
  - Arousal (calm–excited),
  - Dominance (low–high).
3. Brainwave activity was measured in seven frequency bands, which was registered by multichannel EEG equipment from Neuvo SynAmps2 System (Neuvo EEG 64ch, Compumedics, Australia) in the three experimental conditions: Control, 2D, and 3D. EEG recordings were obtained with the standard Ag/AgCl 64-channel scalp electrodes, referenced to the Cpz, and an AFz ground was used. EEGs were continuously recorded at a sampling frequency of 1000 Hz and were bandpass filtered from 0.5 Hz to 40 Hz, and the interelectrode impedances were kept below 5 kO.

In order to process the EEG signals, the open source toolbox EEGLAB was used. The Independent Component Analysis (ICA) was then carried out using infomax algorithm to detect and remove components due to eye movements, blinks, and muscular artifacts. It turned out that there was no considerable increase in artifacts compared to the regular EEG registration (i.e., without a virtual reality helmet).

The spectral power was computed using a Hanning window and fast Fourier transform (FFT) and was averaged across 30 2-s epochs within the classical frequency bandwidth Delta (1–4 Hz), Theta (4–7 Hz), Alpha1 (7–10 Hz), Alpha2 (10–13 Hz), Beta1 (13–20 Hz), Beta2 (20–30 Hz), Gamma (30–40 Hz). Frequency band-power features were computed in the three experimental conditions separately (Control, 2D and 3D).

The statistical analysis was done using Statistica 13.3. We used non-parametrical analysis methods: Mann-Whitney test for between-group comparison, Wilcoxon test for comparing the dependent variables, and Spearman’s rank correlation coefficient for the correlations.

The hypotheses in our study were significant effects of the experimental conditions’ parameters (perception of 2D and 3D natural stimuli) on the subjects’ psycho-emotional state, namely that positive emotion increases and arousal decreases due to perception of natural stimuli in nature-based environment. These effects are presumably enhanced along with an increase in EID.

## 4. Results

### 4.1. The Psychometric Data Analysis

The mean environmental identity index (EID) amounted to 63.2 (SD = 14.0), which is lower than the scale average of 72, thus suggesting decreased connection to the natural environment in the subjects. The Mann–Whitney test revealed a significant difference (U = 20.0, Z = 2.229, *p* = 0.025) in the Index between men (56.6) and women (69.7).

Table 1 presents the means and SDs for the emotional state components per the experimental conditions and the genders. The only gender-related difference trending towards significance (Mann–Whitney *p* = 0.08) was found for Valence in 2D, with more positive self-assessment of the component in men compared to women. The differences between the experimental conditions presented in the table are calculated using Friedman ANOVA. The pair-wise differences are indicated by * and # signs if significant according to the Wilcoxon test.

The data presented in Table 1 suggest that the perception of the natural environment in 2D is accompanied by positive shift of the mood for men, who also demonstrated increased Arousal and Dominance in the 3D condition. For women, significant increase of Arousal and Dominance in 3D can be noted, which was not significantly different from the 2D condition. In this gender group, Valence was also higher in 3D, but the difference was not significant.

**Figure 4:**
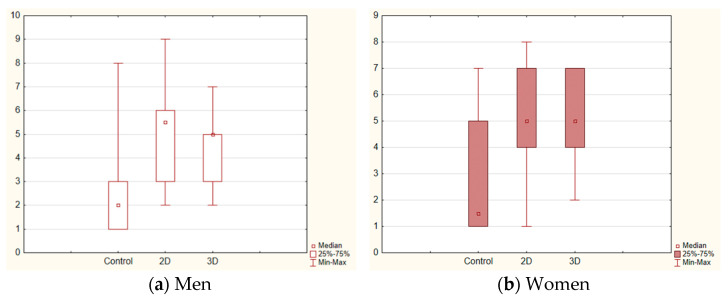
The self-assessments of Arousal in the different experimental conditions for men (**a**) and women (**b**).

In Figure 4, we illustrate the changes in Arousal for men (a) and women (b). It appears that the general effect on Arousal is due to the novel situation—the immersion to the Virtual Reality environment, which causes stress in the first phases of the experience [53].

**Figure 5:**
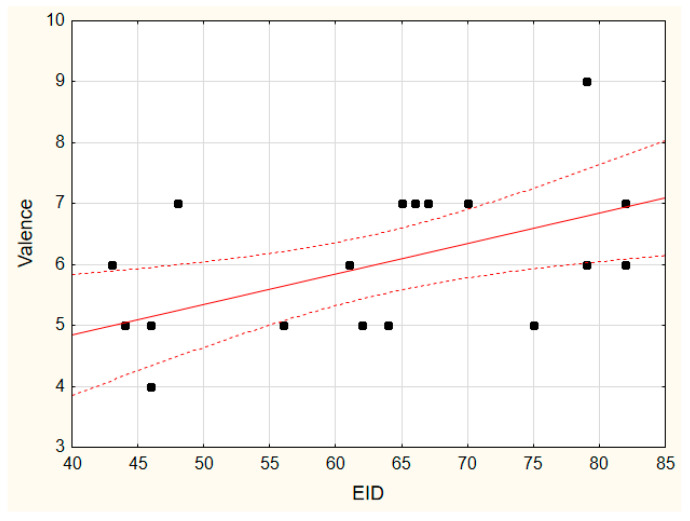
The relationship between the EID and Valence in the 2D condition.

For the EID index, we found significant positive correlations with Valence in 2D (Rs = 0.58, *p* = 0.008, see Figure 5). The effect was prominent in women, who also demonstrated positive correlation with Arousal in 2D (Rs = 0.63, *p* = 0.05). For the men, we did not find a significant correlation between EID in 2D with any of the three emotional state self-assessment components. For the 3D condition, we found significant correlations between the EID index in women with Valence and Arousal (0.63 < Rs < 0.76, 0.01 < *p* < 0.05).

Thus, the observed improvement in the emotional state (Valence) when watching virtual nature in 2D corresponds to the preference of the natural environment according to the “Environmental Identity” questionnaire. However, in women, this effect was accompanied by the significant increase in Arousal, which seemingly suggests a summation of the relaxation effects in the natural environment but activation due to the virtual reality immersion. Hence, the disagreements regarding the effect of natural environment on the subjects’ emotional state noted in various research works (e.g., [4,38]) might be due to not only the gender factor, which shapes spatial orientation and emotional reactivity when processing visual information [24,25,54], but also due to the individual differences in the natural environment attractiveness (EID).

### 4.2. The EEG Data Analysis

**Figure 6:**
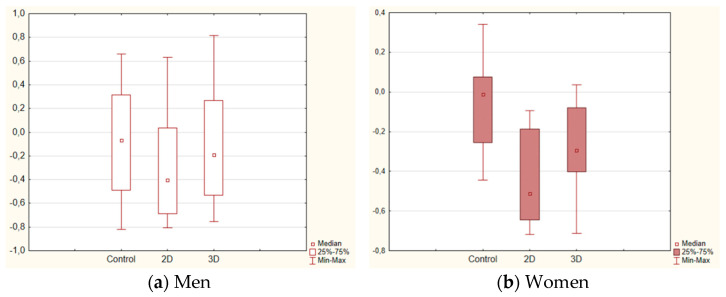
The changes in Alpha2 EEG power in the different experimental conditions for men (**a**) and women (**b**).

The ln-transformed EEG power indices were analyzed in the seven frequency bands using non-parametric ANOVA for each group with the subsequent comparison between the three experimental conditions (Control, 2D, and 3D). The analysis results for the bands with the group-specific changes (Alpha2 and Beta2) are presented in Table 2. Figure 6 demonstrates the similar decreases in Alpha2 power for men and women in 2D and 3D conditions (with greater variability in men) and the significantly higher power in 3D compared to 2D.

The experimental conditions had a highly significant effect on EEG in Delta, Theta, Alpha1, Beta1, and Gamma bands (see in Table 3), but it was independent of the gender factor.

Our correlations analysis of EEG brain activity did not discover any significant relationships between the signal power and the emotional state self-assessments, in either 2D or 3D. However, we found significant correlations between the EID and the EEG power in high-frequency Beta and Gamma bands: positive one for women in both 2D and 3D (0.64 < Rs < 0.74, 0.01 < *p* < 0.05), but negative one for men (−0.66 < Rs < −0.72, 0.02 < *p* < 0.04), and only in 2D.

## 5. Discussion and Conclusions

The results obtained in our study suggest virtual nature presentation-related effect of the cerebral cortex activation in the 2D condition, registered as the power decrease in both low- and high-frequency bands. Their increase in the 3D condition compared to 2D, which was registered for all the frequency bands, might reflect not only adaptation to the experimental environment, but also the effect of relaxation associated with the virtual immersion in the natural environment.

The relaxation effect observed with the exposure to the nature-related images in both 2D and 3D formats has been found for various psychophysical measures: EEG indexes, and/or heart rate variability [55,56]. However, there are also suggestions of EEG correlates’ meaningfulness only for the negative assessment of the virtual environment [57] or the decrease in alpha rhythm for open eyes, but its increase with closed eyes after staying with nature for many days compared to the urban environment [58]. This effect is seen by the authors as the changes in the neural mechanisms of information selection that are caused by resting with the nature. Correspondingly, the immersion to the natural environment can lead to different results depending on the degree of the relaxation effect caused by the positive mood shift or, on the contrary, by the increase in the brain activation ability due to the recovery of the internal attention resources.

Supposedly, the relaxation effect might explain the disagreement between our results and the previous findings of some other researchers [59], who reported an increase in Beta power in 3D compared to 2D, where they presented the specially tailored positive, neutral, and negative VR video materials. The emotional state self-assessments in our experimental conditions suggest a gradual improvement of the subjects’ mood under the natural environment stimulus presentation (a virtual walk in a forest). In comparison with the Control condition, this effect was significant in 2D for Valence in men, as well as in 3D for all the emotional components except for Valence in women.

The obtained contrasting correlations between EID and the high-frequency EEG oscillations (i.e., positive one for women but negative one for men) might be related to the preference of an allocentric navigational strategy in women [25,26], which is accompanied by increased attention towards various natural environment-related stimuli. Because of the more pronounced egocentric strategy of orientation in space in men, higher EID values together with lower high-frequency EEG powers likely reflect the lower engagement of less visual attention resources in the natural stimuli processing. Another factor for the individual diversity of reactions towards the modeled 2D or 3D natural environment could be the summation of the relaxation effect caused by the biophilic design of the stimuli (hence, the increase in the alpha rhythm power) [55,60] and the brain activation (the increase in the beta rhythm power) because of the emotional reaction to the novelty of the virtual reality immersion and/or increase in the visual attention volume and the automated motor action plans activated by objects (affordances) in the 3D condition [21,22,59].

We can conclude that the subjective assessment indexes and the EEG powers were similar for both men and women when immersed in the virtual natural environment. The most pronounced effect of the gender factor was found in the relation between the individual environmental identity (EID) and the EEG powers in the high-frequency Beta and Gamma bands. The observed contrast is likely due to the different spatial orientation strategies in men and women and different emotional reactions to a natural environment. The relaxation effect found in Alpha2 band in the 3D condition, which was more pronounced in men (0.08 ± 0.57) than in women (−0.29 ± 0.23), can be related to the higher pace of VR immersion in the former group due to the diversity of the signal’s spatial processing strategies. The discovered cerebral cortex activation in 2D, according to the biopotentials’ power in both low- and high-frequency bands, reflects the wide range of the brain functional systems’ resources engaged in the visual information processing. The weakening of this effect in the 3D condition confirms the capability to further increase the relaxation and improve the mood when immersed in the VR natural environment compared to the conventional 2D natural stimuli presentation.

Limitations of our study include a relatively small sample with low age diversity. We also used rather basic and short stimuli, as well as a short period of EEG registration during 2D and 3D conditions, so the validity of the results needs to be further explored using a prolonged timeframe. In addition, we did not alter the order of the stimuli presentation in 2D and 3D conditions, which might have led to habituation in the subjects. However, their small number and the different individual reactivity to the stimuli were arguably more notable than the habituation effect. Therefore, our plans for future research include a more detailed study of the frequency–spatial differences in the subjects’ cerebral cortex activity when immersed in nature-related virtual content with a larger sample incorporating greater diversity in age.

## Figures and Tables

**Table 1:** Virtual nature stimulus-related changes in the emotional state self-assessment components in the gender groups (mean ± SD).

		Men			Women	
	Valence	Arousal	Dominance	Valence	Arousal	Dominance
**Control**	5.0 ± 1.1 *	2.8 ± 2.4 *	3.6 ± 2.2 *	5.6 ± 1.3	3.0 ± 2.4 *	3.5 ± 2.2 *
**2D**	6.4 ± 0.8 *	3.3 ± 1.3 ^#^	4.1 ± 1.4	5.6 ± 1.5	4.0 ± 1.9	4.7 ± 2.2
**3D**	6.3 ± 1.5	4.6 ± 1.6 *^#^	5.2 ± 2.2 *	6.4 ± 1.2	5.1 ± 1.9 *	5.1 ± 2.7 *
Difference	Χ(2) = 6.81, *p* = 0.03	Χ(2) = 7.00, *p* = 0.03	Χ(2) = 4.06, *p* = 0.13	Χ(2) = 1.87, *p* = 0.39	Χ(2) = 5.61, *p* = 0.06	Χ(2) = 5.36, *p* = 0.07

* indicates significant difference (*p* < 0.05) between an experimental condition and Control; # indicates significant difference (*p* < 0.05) between the 2D and 3D conditions (Wilcoxon test).

**Table 2:** Virtual nature stimulus-related changes in the EEG power (two frequency bands) in the gender groups (mean ± SD).

	Men		Women	
	Alpha2	Beta2	Alpha2	Beta2
**Control**	−0.09 ± 0.53 *	−0.72 ± 0.26 *	−0.07 ± 0.25 *	−0.86 ± 0.20
**2D**	−0.29 ± 0.49 *^#^	−0.83 ± 0.25 *^#^	−0.44 ± 0.23 *	−0.91 ± 0.27 ^#^
**3D**	−0.08 ± 0.57 ^#^	−0.63 ± 0.30 ^#^	−0.29 ± 0.23	−0.78 ± 0.26 ^#^
Difference	Χ(2) = 6.21 *p* = 0.05	Χ(2) = 5.03 *p* = 0.08	Χ(2) = 10.42 *p* = 0.006	Χ(2) = 3.83 *p* = 0.15

* indicates significant difference (*p* < 0.05) between an experimental condition and Control; # indicates significant difference (*p* < 0.05) between the 2D and 3D conditions (Wilcoxon test).

**Table 3:** Virtual nature stimulus-related changes in EEG power in the five frequency bands (mean ± SD).

	Delta	Theta	Alpha1	Beta1	Gamma
**Control**	0.484 ± 0.417 *	−0.100 ± 0.308 **	−0.126 ± 0.349 ***	−0.540 ± 0.277 **	−1.036 ± 0.210 *
**2D**	0.316 ± 0.312 *^###^	−0.280 ± 0.255 **^###^	−0.401 ± 0.291 ***^###^	−0.693 ± 0.249 **^##^	−1.085 ± 0.228 ^##^
**3D**	0.525 ± 0.416 ^###^	−0.116 ± 0.328 ^###^	−0.214 ± 0.373 ^###^	−0.561 ± 0.319 ^##^	−0.903 ± 0.250 *^##^
Difference	Χ(2) = 12.4 *p* = 0.002	Χ(2) = 17.5 *p* = 0.0002	Χ(2) = 27.1 *p* < 0.0001	Χ(2) = 10.9 *p* = 0.004	Χ(2) = 9.7 *p* = 0.008

* *p* < 0.05, ** *p* < 0.01, *** *p* < 0.001 indicate significant differences between an experimental condition and Control; ## *p* < 0.01, ### *p* < 0.001 indicate significant differences between the 2D and 3D conditions (Wilcoxon test).

## Data Availability

The data presented in this study are available on request from O.R. (the second author). The data are not publicly available due to privacy reasons.
